# Bacterial magnetosomes as an efficient gene delivery platform for cancer theranostics

**DOI:** 10.1186/s12934-017-0830-6

**Published:** 2017-11-28

**Authors:** Qinglei Dai, Ruimin Long, Shibin Wang, Ranjith Kumar Kankala, Jiaojiao Wang, Wei Jiang, Yuangang Liu

**Affiliations:** 1 College of Chemical Engineering, Huaqiao University, Xiamen, 361021 People’s Republic of China; 2 Institute of Pharmaceutical Engineering, Huaqiao University, Xiamen, 361021 People’s Republic of China; 3 Fujian Provincial Key Laboratory of Biochemical Technology, Huaqiao University, Xiamen, 361021 People’s Republic of China; 4 State Key Laboratories for Agrobiotechnology and College of Biological Sciences, China Agricultural University, Beijing, 100094 People’s Republic of China

**Keywords:** Bacterial magnetosomes, siRNA, Polyethylenimine, Gene therapy

## Abstract

**Background:**

Gene therapy has gained an increasing interest in its anti-tumor efficiency. However, numerous efforts are required to promote them to clinics. In this study, a novel and efficient delivery platform based on bacterial magnetosomes (BMs) were developed, and the efficiency of BMs in delivering small interfering ribonucleic acid (siRNA) as well as antiproliferative effects in vitro were investigated.

**Results:**

Initially, we optimized the nitrogen/phosphate ratio and the BMs/siRNA mass ratio as 20 and 1:2, respectively, to prepare the BMs–PEI–siRNA composites. Furthermore, the prepared nanoconjugates were systematically characterized. The dynamic light scattering measurements indicated that the particle size and the zeta potential of BMs–PEI–siRNA are 196.5 nm and 49.5 ± 3.77 mV, respectively, which are optimum for cell internalization. Moreover, the confocal laser scanning microscope observations showed that these composites were at a proximity to the nucleus and led to an effective silencing effect. BMs–PEI–siRNA composites efficiently inhibited the growth of HeLa cells in a dose-as well as time-dependent manner. Eventually, a dual stain assay using acridine orange/ethidium bromide, revealed that these nanocomposites induced late apoptosis in cancer cells.

**Conclusions:**

A novel and efficient gene delivery system based on BMs was successfully produced for cancer therapy, and these innovative carriers will potentially find widespread applications in the pharmaceutical field.

## Background

Cancer is one of the leading causes of deaths, accounting for millions of deaths annually. More often, chemotherapy is the primarily advised therapeutic regimen after surgery and/or radiation therapy to improve the survival rate in patients with cancer. However, most of the chemotherapeutic agents result in several adverse effects due to their non-specific uptake by healthy cells, poor bioavailability and multidrug resistance (MDR) attained by cancer cells, among others [1]. In addition, these undesired effects result in the lower therapeutic efficacy of conventional chemotherapeutic agents. To this end, gene therapy has shown a great potential in the treatment of many cancers because of the ability of genes in eradicating the hereditary diseases and replace the defective cell specifically [2, 3]. Moreover, the small (or short)-interfering ribonucleic acid (siRNA), often called as silencing RNA, is a class of chemically synthesized double-stranded RNA molecules with 19–23 nucleotides that can trigger the silencing of homologous gene expression [4]. Inspired by this fact, the researchers have harnessed the siRNA for various applications in biomedical field [5, 6]. However, there has not been an anticipated success for their exploration in clinics, due to various reasons such as lack of stability in organism caused by ribonuclease (RNase) degradation, poor cellular uptake, and endosomal trapping, among others [7]. To overcome these issues, a wide-variety of non-viral vectors have been used to deliver siRNA, including lipid, cationic polymers, and inorganic nanoparticles, which are advantageous over virus-based vectors.

Out of various non-viral vectors available, cationic polymers have gained the significant importance for the efficient conveyance of genes due to their advantages such as high stability in physiological fluids, controlled release of active pharmaceutical agents (APIs) including genes, large capacity of gene packing and ease of structural modification to improve the transfection efficiency and stability of genes. Polyethyleneimine (PEI) is one of the most promising polymeric substrates explored for the efficient delivery of DNA [8, 9], siRNA [10, 11], and oligonucleotides [12]. More often, the genes with desired nucleotide sequences are encapsulated in the PEI through electrostatic interactions. In addition, the interesting feature of PEI is that it condenses the anionic siRNA and subsequently protects the siRNA from degradation by RNase [13]. Preceding research has indicated that PEI can prevent exocytosis through the proton sponge effect, which induces the flow of chloride ions and thereby promotes the osmotic swelling of endosomes/lysosomes and subsequently releases the APIs [14]. Despite its efficiency in delivery, several factors of PEI significantly affect the transfection efficiency and toxicity of genes such as the molecular weight and structure of PEI. In addition, advancements in the PEI-based design are still obligatory to achieve the efficient delivery of genes by reducing the adverse effects simultaneously.

Pharmaceutical carriers often use polymers, dendrimers, micelles, liposomes, inorganic nanomaterials and so on [15–21], which can all be employed for drug delivery system. Amongst inorganic nanomaterials, bacterial magnetosomes (BMs) have shown a great potential as a novel carrier due to their excellent biocompatibility, high surface area to volume ratio, superparamagnetism and abundant active sites on the membrane of BMs [22]. More often, BMs are extracted from magnetotactic bacteria with magnetic iron oxide or iron sulfide enclosed by a natural phospholipid membrane [23], which endowed them with high biocompatibility. In a case, the purified and sterilized BMs have shown that they were non-toxic to mouse fibroblasts in vitro [22]. In addition, the pyrogen test revealed that the administered BMs (1 mg) resulted in no significant change in the body temperature of rabbits. In another study, Sun et al. evaluated the acute toxicity, immunotoxicity, and cytotoxicity of BMs [24]. The blood examination results of BMs have shown no significant effect compared to the control group of rats. However, BMs showed a slight cytotoxicity in H22, HL60, or EMT6 cell lines. In recent times, BMs have gained an increasing interest for the delivery of proteins, chemotherapy drugs and DNA [25–27].

Motivated by these facts, this study reports the synthesis of gene delivery system based on BMs for the effective delivery of siRNA by using PEI as a crosslinker (BMs–PEI–siRNA). Furthermore, various techniques were used to systematically characterize the nanocomposites such as transmission electron microscope (TEM) for morphology, DLS measurements for particle size distribution and others. Furthermore, the stability and bioactivity studies were performed to elucidate the integrity and the anti-proliferative effects of the siRNA-loaded BMs, respectively.

## Results and discussion

### Characterization of BMs and its conjugates

**Fig. 1:**
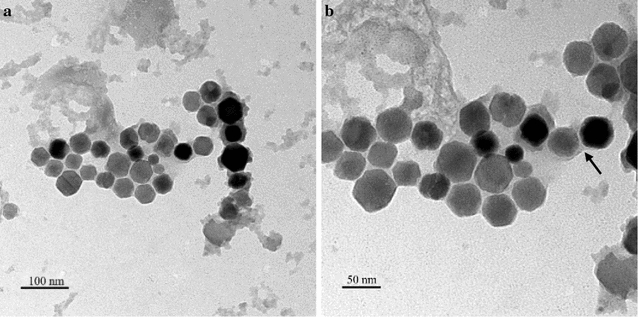
TEM images of BMs (black arrows indicating the uniform and clear lipid membrane of BMs)

From the TEM images (Fig. 1), it is evident that the particle sizes and morphology of BMs are uniform and the diameter was between 30 and 50 nm with the hexagonal arrangement (Fig. 1a). It reflects the coated layer over the BMs indicating that the distinct membrane was composed of phospholipids and fatty acids [28]. Furthermore, the zeta potential value of BMs was measured by adjusting the pH of the sample to physiological pH (7.4). The surface charge of BMs was − 48.3 ± 2.6 mV, owing to the existence of abundant lipids and amino groups [29]. However, the negative surface charge is countered with a positively-charged PEI for the efficient loading of genes, which condenses the anionic siRNA molecules, and interacts with negatively-charged BMs via electrostatic interactions.

**Fig. 2:**
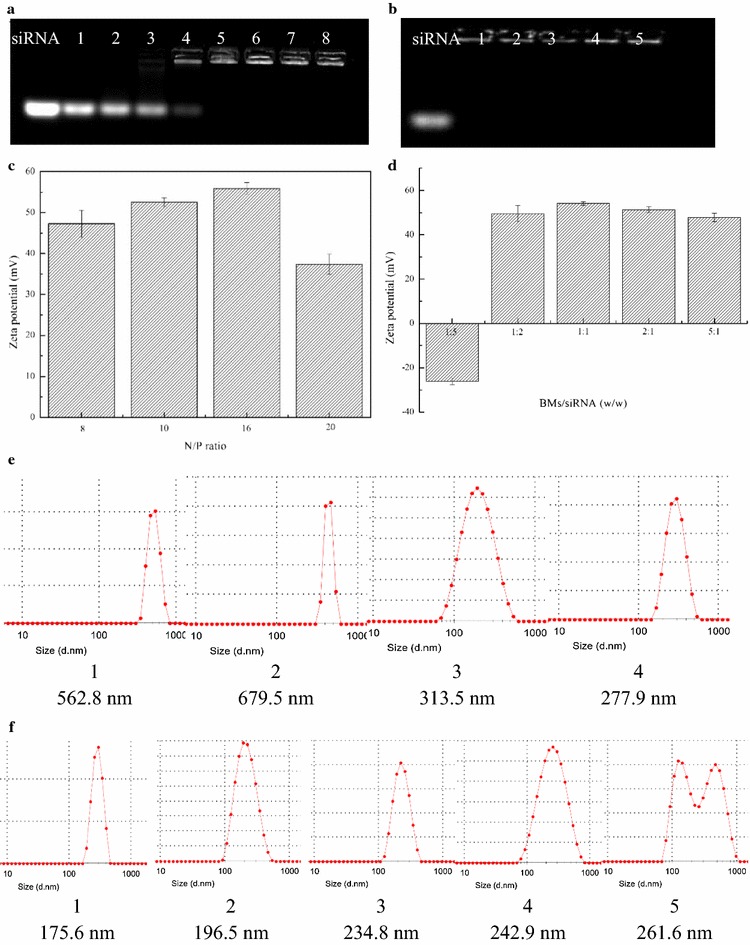
Physical characterization of BMs–PEI–siRNA nanocomposites including the agarose gel electrophoresis results for optimization of formulation. Images showing the gel electrophoresis results of BMs–PEI–siRNA composites **a** at different N/P ratios, 1–8: 0, 1, 2, 4, 8, 10, 16, 20; and **b** at different BMs/siRNA weight ratios, 1–5: 1:5, 1:2, 1:1, 2:1, 5:1 along with free siRNA; zeta potential values of BMs–PEI–siRNA composites **c** at different N/P ratios; and **d** at different BMs/siRNA mass ratios; hydrodynamic diameters of BMs–PEI–siRNA composites **e** at different N/P ratios, 1–4: 8, 10, 16, 20; **f** at different BMs/siRNA ratios, 1–5: 1:5, 1:2, 1:1, 2:1, 5:1

To optimize the formulation of BMs–PEI–siRNA, we performed agarose gel electrophoresis of samples with different N/P ratios concerning the nitrogen in PEI and phosphates in siRNA respectively, and the results were shown in Fig. 2a. The N/P ratio at which the undetected fraction of free siRNA demonstrates that the siRNA was successfully bound to PEI via electrostatic interactions. The retardation efficiency was increased with the increase in N/P ratio. Eventually, the optimized N/P ratio was found above 8, and the samples of BMs–PEI–siRNA composites were prepared at that ratio and systematically characterized using various techniques.

The dynamic light scattering (DLS) measurements gave the hydrodynamic mean diameters as well as zeta potential values of BMs–PEI–siRNA composites. As shown in Fig. 2c, e, the diameters of the synthesized nanocomposites decreased with the increase of N/P ratios, demonstrating that the addition of siRNA and PEI complex resulted in the compact nanocomposites. Moreover, the zeta potential values of respective nanocomposites were in a positive range, which confirms the conjugation of PEI. The hydrodynamic diameter and the positive potential of BMs–PEI–siRNA formulation with N/P ratio 20 (200 nm) are optimum for the accumulation through enhanced permeation and retention (EPR) effect and ease of cellular internalization in the tumor [30].

Further, we investigated the concentration of BMs that would be effective in the formation of BMs–PEI–siRNA through agarose gel electrophoresis. The concentration of BMs at which the gel devoid of free siRNA band indicates that they have no significant effect on the immobilization. Figure 2b shows the gel images of BMs–PEI–siRNA composites with different BMs/siRNA weight ratios, at which the bandwidth of BMs/siRNA weight ratio 1:2 was found optimum. Moreover, the size was relatively small (Fig. 2f) and the potential was positive (Fig. 2d), at this ratio, which was extremely beneficial for establishing the interactions with the cell and subsequent internalization process.

### Stability studies

**Fig. 3:**
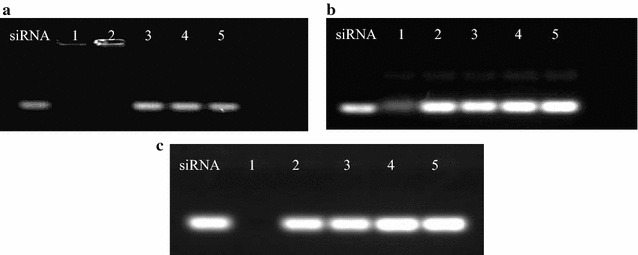
Agarose gel electrophoresis assay of BMs–PEI–siRNA composites. **a** The heparin decomplexation assay. 1: Naked siRNA; 2–5: BMs–PEI–siRNA at different heparin/siRNA weight ratio: 2, 10, 25, 100, respectively; **b** the serum stability, 1: naked siRNA incubation with 50% FBS for 60 min; 2–5: BMs–PEI–siRNA incubation with 50% FBS for 60, 90, 120, 150 min, respectively; **c** the enzyme stability of BMs–PEI–siRNA: 1: naked siRNA incubation with RNase A for 60 min; 2–5: BMs–PEI–siRNA incubation with RNase A for 60, 90, 120, 150 min, respectively

siRNA is one of the most sensitive biomolecules in the body, which suffer from certain limitations during delivery such as short circulation times, reduced therapeutic effects and others due to in vivo degradation [31]. In addition, a few factors such as serum proteins are considered during the formulation of genes for cancer theranostics. Herewith, we demonstrated the stability of our design through various methods such as decomplexation assay, enzyme stability assay, and others. In heparin decomplexation assay, the siRNA dissociates from the synthesized nanocomposites due to the stronger interaction of the heparin with the nanocomposites. The experiment was performed by mixing various concentrations of heparin with the nanocomposites, and the resultant siRNA in the supernatant was subjected to gel electrophoresis. The results (Fig. 3a) indicated that the siRNA was utterly dissociated from the nanocomposites at a specific weight ratio of heparin to siRNA (10:1) after incubating for 15 min. Figure 3b elucidates the stability of siRNA in the presence of serum proteins. It is evident that the naked siRNA was degraded rapidly in 50% FBS for 60 min, while the fraction can be still observed in the case of BMs–PEI–siRNA nanocomposites after 150 min of exposure, demonstrating that the designed nanocomposites offered significant protection to siRNA. Eventually, the stability of siRNA in the presence of enzymes was demonstrated by suspending the designed nanocomposites in the presence of RNase A. The results showed that the immobilization of siRNA in the PEI network on BMs significantly reduced the degradation, which can enhance the circulation time in vivo.

#### Cell viability assay

**Fig. 4:**
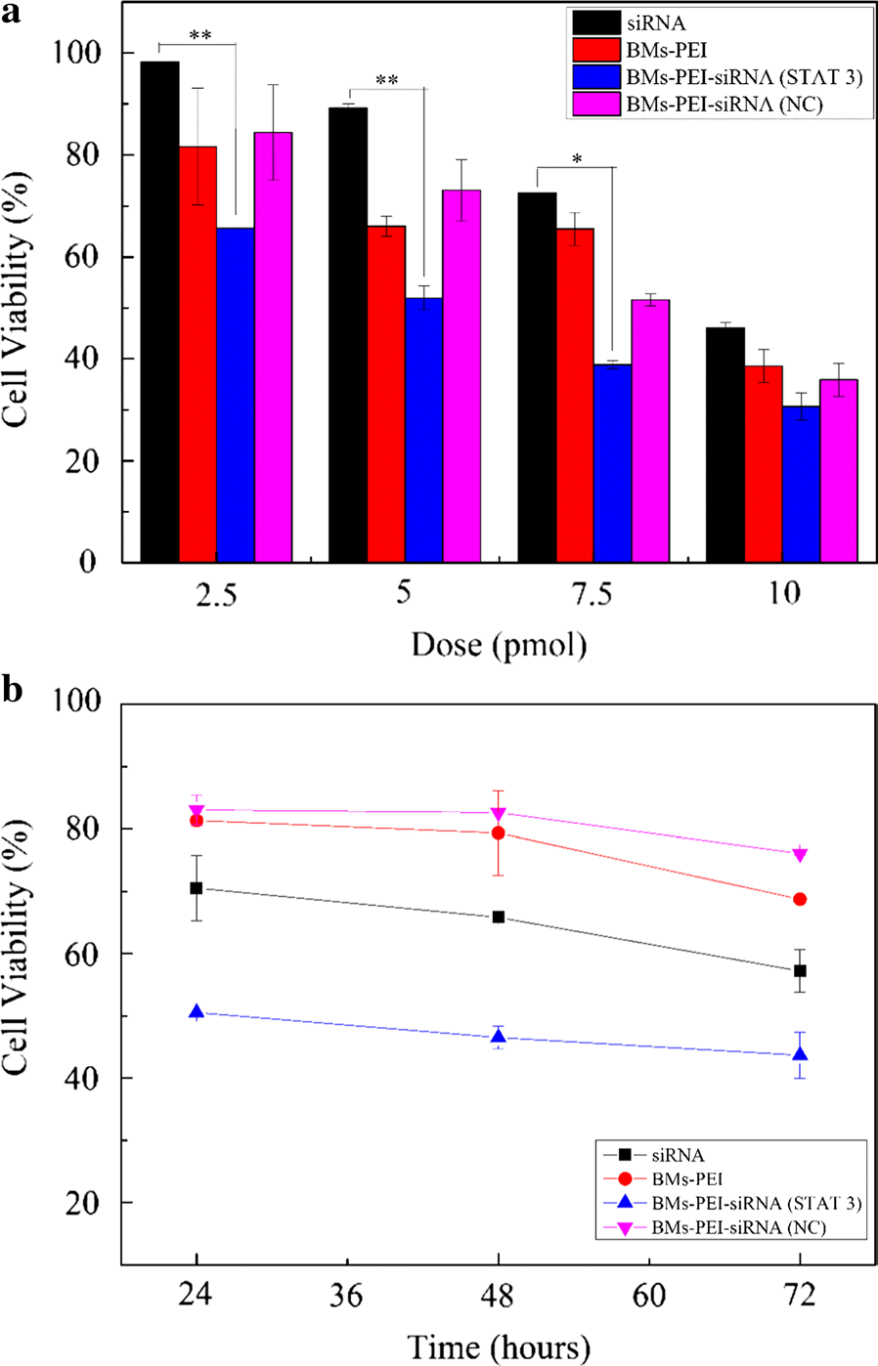
Cytotoxic effects of BMs–PEI–siRNA nanocomposites. **a** Dose-dependent inhibition and **b** time-dependent inhibition of siRNA, BMs–PEI, BMs–PEI–siRNA (STAT 3) and BMs–PEI–siRNA (NC) on HeLa cells (*p < 0.05; **p < 0.01)

The anti-tumor efficacy of our novel BMs-based gene delivery system was performed using CCK-8 assay in the HeLa cell line. The experiments were designed such that they represent both dose-dependent by treating various doses and time-dependent cytotoxicity by measuring the viability at different time points. In the dose-dependent assay, the viability of cells gradually decreased with the increase in the concentration of nanocomposites (Fig. 4a). Moreover, the inhibition rate of BMs–PEI–siRNA (STAT 3) composites at a dose of 10 pmol was significantly higher than that of siRNA alone accounting for 70% of cell death. The inhibition effect of BMs–PEI–siRNA (NC) was similar to that of BMs–PEI vector, indicating that the cytotoxicity of siRNA was sequence-specific. Based on these results, the nanoconjugates at a concentration of 5 pmol were chosen as an optimized dose for further investigations.

As shown in Fig. 4b, BMs–PEI, and BMs–PEI–siRNA (NC) showed no apparent growth inhibition of cells within the incubation times, indicating that the BMs–PEI composites resulted in low toxicity. Cytotoxicity of BMs–PEI–siRNA (STAT 3) sample showed the time-dependent inhibitory effect on HeLa cells about 40% after 72 h of incubation and was significantly higher compared to that of siRNA treatment group of cells.

#### Cell apoptosis assay

**Fig. 5:**
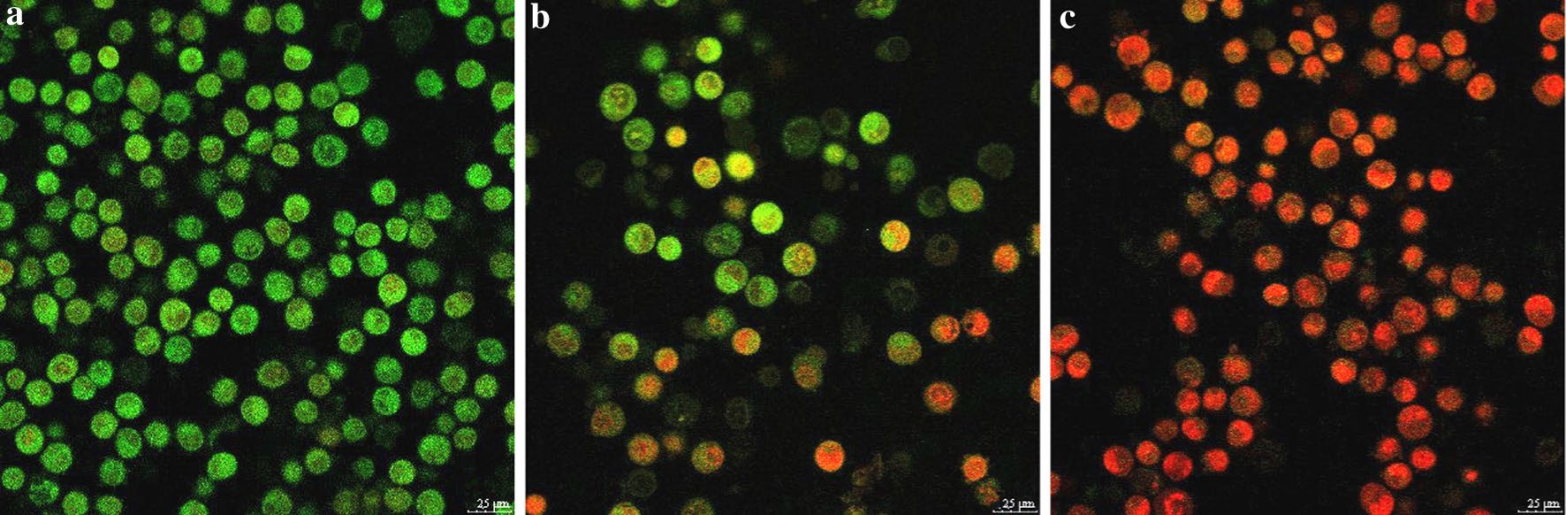
AO/EB dual staining of HeLa cells after culturing for 48 h. **a** Control (media alone); **b** naked siRNA; **c** BMs–PEI–siRNA

To further assess the anti-tumor effect of BMs–PEI–siRNA composites, the cell apoptosis of designed sample was examined using acridine orange/ethidium bromide (AO/EB) dual stain. As shown in Fig. 5, cells in the negative control group were green in color elucidating no apparent cell apoptosis. Comparatively, the cells in siRNA, as well as BMs–PEI–siRNA treated groups indicated more orange stained cells demonstrating that the cells underwent early and late apoptosis. The results were consistent with that of anti-tumor efficacy. Overall, siRNA-loaded BMs–PEI delivery system not only efficiently expressed the silencing effect of siRNA but also induced the apoptosis compared to the naked siRNA.

### Cellular uptake of BMs–PEI–siRNA nanocomposites

**Fig. 6:**
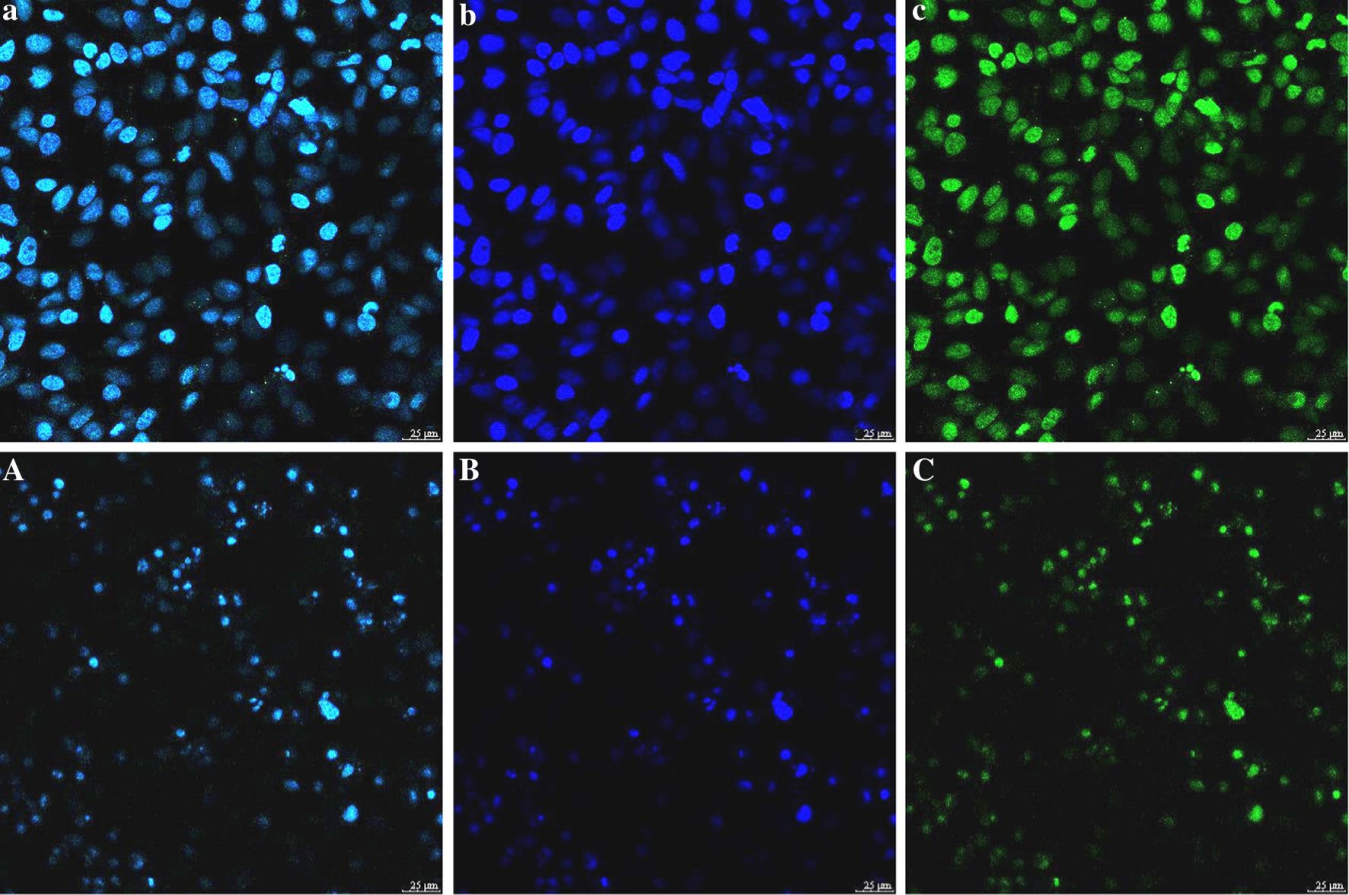
CLSM images illustrating the cellular uptake of our designed nanocomposites (**a**–**c** 0.5 h, **A**–**C** 6 h)

Indeed, cellular uptake of nanoparticles plays a crucial role during the formulation of nano-delivery systems for the efficient delivery of therapeutic cargo. However, the positively-charged BMs–PEI nanoparticles are highly suitable for delivering siRNA as they interact with the negatively-charged cell membrane. To validate the internalization of designed nanocarriers, we labeled FAM to siRNA and tracked the presence of nanocomposites using confocal laser scanning microscope (CLSM) in HeLa cells after incubation for 0.5 and 6 h (Fig. 6). Interestingly, the BMs–PEI–siRNA nanocomposites were at the proximity of the nucleus in cells. In addition, some characteristic changes associated with the apoptosis such as chromatin condensation and nucleus shrinkage were observed in the cells treated with BMs–PEI–siRNA nanocomposites, indicating that the cells underwent apoptosis. This phenomenon was different from the results of cell uptake in most previous studies [32, 33]. Preceding reports indicated that the RNA interference (RNAi) occurs in the cytoplasm, while other studies have revealed that potent RNAi expressed in the nucleus of human cells [34]. The reason for these contrast findings might be the altered dynamics and distribution of siRNA due to the presence of BMs, which promoted their delivery close to the nucleus.

## Conclusions

In summary, we designed a novel delivery system based on siRNA-loaded BMs using cationic PEI as a crosslinker. After improving the synthetic conditions, the optimal BMs–PEI–siRNA nanocomposites have shown an enhanced cellular uptake and exhibited serum stability as well as enzymatic hydrolysis. These stable nanocomposites resulted in more significant inhibitory effects on HeLa cells. This delivery system takes advantage of efficient delivery of siRNA into cancer cells, and also provides an opportunity for the development of various novel therapeutic strategies.

## Experimental section

### Materials

BMs extracted from *Magnetospirillum gryphiswaldense* MSR-1 were presented kindly by professor Li Ying and Jiang Wei (Department of Microbiology, China Agricultural University). STAT 3 siRNA and siRNA (NC) were purchased from GenePharma Co., Ltd. (Shanghai, China), siRNA (NC) was used as the negative control of STAT 3 siRNA without homology. Branched PEI (MW: 25 KD) was purchased from Sigma Aldrich (USA). The cervical carcinoma cell line (HeLa cells), was obtained from China Academy Typical Culture Preservation Committee Cell Library (Shanghai, China). Cell culture medium was composed of Dulbecco’s Modified Eagle’s Medium (DMEM) supplemented with 10% fetal bovine serum (FBS). The cells were incubated in humidified air maintained at 37 °C with 5% CO_2_.

### Preparation and characterization of BMs

The extraction process of BMs was performed by following our reported procedure given below [35]. Microbial cells of *M. gryphiswaldense* MSR-1 were suspended in phosphate buffered saline (PBS, 0.1 M, pH 7.4) and then the cell membrane was disrupted by ultrasonication. The cell debris was removed by magnetic adsorption, and the process was repeated for about 20 times. The resultant suspension of BMs was treated with DNase I for 2 h at 37 °C. The BMs were then washed for about 20 times and conserved at − 20 °C after being freeze-dried. Further, the suspension of BMs was subjected to physical characterization.

The morphology of BMs was confirmed by capturing images using TEM. The zeta potential and particle size distribution of BMs were measured by Zetasizer (ZEN3600, Malvern Instruments Ltd, UK).

### Preparation of BMs–PEI–siRNA nanocomposites

**Fig. 7:**
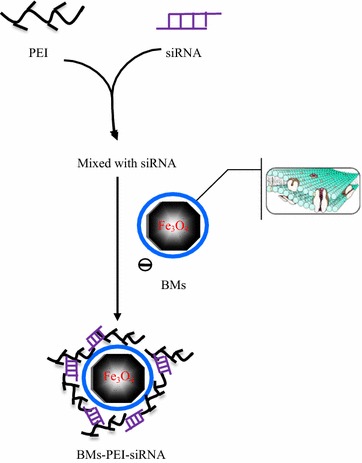
Schematic illustration showing the synthetic outline of BMs–PEI–siRNA

The nanocomposites with different nitrogen of PEI/phosphate of siRNA (N/P) ratios (N/P ratios were set as 0, 1, 2, 4, 8, 10, 16 and 20) were prepared by mixing a certain amount of siRNA with PEI in diethyl pyrocarbonate (DEPC) water, and fixed amounts of BMs were added (BMs/siRNA mass ratio was 1:2), followed by vortexing for 2 min and incubated for 25 min at room temperature. The synthetic process of the composites was demonstrated in Fig. 7.

To obtain the optimal weight ratio of BMs to siRNA, various amounts of BMs were added to the PEI/siRNA (N/P = 20) complexes in DEPC water (weight ratios of BMs to siRNA were set as 1:5, 1:2, 1:1, 2:1 and 5:1), and incubated for 25 min to obtain BMs–PEI–siRNA nanocomposites. The binding ability was estimated by agarose gel retardation assay.

### Electrophoresis assay

The agarose gel electrophoresis assay was performed to estimate the encapsulation efficiency of siRNA in BMs–PEI–siRNA nanocomposites. The resultant nanocomposites were loaded on 0.8% (w/v) agarose gel containing 1% (v/v) Gel Stain in tris acetate EDTA (TAE) buffer, and the gel was run at 70 V for 20 min. The gel image was captured using UV transilluminator and a digital imaging system (GIS2008, Tanon Science & Technology Co., Ltd, China).

### Stability studies

The stability of designed nanocomposites was determined by incubating them in various conditions provided, which mimic the physiological fluids. One of them was the heparin decomplexation assay. Heparin (heparin/siRNA weight ratios: 2, 10, 25 and 100) was mixed with BMs–PEI–siRNA and incubated for another 15 min at room temperature. The resultants were subjected to agarose gel electrophoresis (DYY-6C, Liuyi Biological Technology Co., Ltd, China).

To determine the serum stability assay, naked siRNA and BMs–PEI–siRNA nanocomposites were treated with 50% fetal bovine serum and incubated for 60, 60, 90, 120 and 150 min. At predetermined time intervals, heparin was added to nanocomposites group, followed by incubation for 15 min. All samples were loaded on 0.8% agarose gel electrophoresis for retardation analysis.

Further, the enzyme stability assay was performed by incubating naked siRNA and BMs–PEI–siRNA nanocomposites individually with RNase A for 60, 60, 90, 120 and 150 min. The samples were then subjected to agarose gel electrophoresis.

#### Cell viability assay

The cytotoxicity of the designed nanoconjugates was measured using CCK-8 assay at a different siRNA concentration (dose-dependent) and incubation times (time-dependent). HeLa cells were seeded into 96-well plates at 2 × 10^4^ cells/well and incubated for proper cell attachment. After 24 h of incubation, the cells were subjected to treatment with siRNA, BMs–PEI, BMs–PEI–siRNA (STAT 3) and BMs–PEI–siRNA (NC) (the contents of siRNA were set as 2.5, 5, 7.5 and 10 pmol) in 100 μL of serum-free DMEM for 6 h. The medium was then replaced with 200 μL of DMEM containing 10% FBS and incubated for further 48 h. At the end of the incubation, 20 μL of CCK-8 reagent was added to each well and further incubated for 2 h. Finally, the absorbance was recorded by using a microplate reader at 450 nm (Multiskan GO, Thermo Scientific Co., Ltd, USA).

Time-dependent assessment of cell viability was performed as described above by incubating the cells with samples [siRNA, BMs–PEI, BMs–PEI–siRNA (STAT 3) and BMs–PEI–siRNA (NC) (the content of siRNA was 5 pmol)] at a different time periods 24, 48 and 72 h.

#### Cell apoptosis assay

To observe the cell apoptosis induced by BMs–PEI–siRNA nanocomposites, HeLa cells were seeded at a density of 1 × 10^5^ cells/well in 24-well plates and incubated for 24 h. Later, cells were treated with siRNA and BMs–PEI–siRNA (the concentration of siRNA was 50 nM) suspended in 0.5 mL serum-free DMEM for 6 h and then replaced with 1 mL DMEM containing 10% FBS and incubated for 48 h. Subsequently, cells were harvested and washed three times with PBS, then 25 μL of cell suspension was stained with 1 μL of AO/EB dual stain reagent for 2–3 min in the dark according to the manufacturer’s instructions. The apoptotic cells were analyzed by observing them under CLSM (Leica TCS SP8, Germany).

### Cellular uptake study

HeLa cells were cultured on 35 mm glass-bottom dishes at a density of 4 × 10^5^ cells/dish and incubated for 24 h for proper cell attachment. Cells were then treated with FAM-labeled BMs–PEI–siRNA nanocomposites (the concentration of siRNA was 50 nM) for 0.5 and 6 h. After pirating the medium, the cells were washed thrice with cold PBS and then the cells were fixed with formaldehyde (4%) for 10 min, then washed and stained with DAPI. The dishes were eventually observed under CLSM (Leica TCS SP8, Germany).

## Abbreviations

AO/EB: acridine orange/ethidium bromide
N/P: nitrogen/phosphate
MDR: multidrug resistance
siRNA: small interfering RNA
RNase: ribonuclease
PEI: polyethyleneimine
APIs: active pharmaceutical agents
DLS: dynamic light scattering
TEM: transmission electron microscope
EPR: enhanced permeation and retention
RNAi: RNA interference
PBS: phosphate buffered saline
BMs: bacterial magnetosomes
BMs–PEI–siRNA: siRNA loaded BMs delivery system by using polyethyleneimine as a crosslinker
DEPC: diethyl pyrocarbonate
TAE: tris acetate EDTA
DMEM: Dulbecco’s Modified Eagle’s Medium
CLSM: confocal laser scanning microscope
